# Supplementary figures and images for: Skeletal dysplasia-causing TRPV4 mutations suppress the hypertrophic differentiation of human iPSC-derived chondrocytes
Source: eLife. 2023 Feb 22;12:e71154. doi: 10.7554/eLife.71154 (PMC9949800; doi:10.7554/eLife.71154)

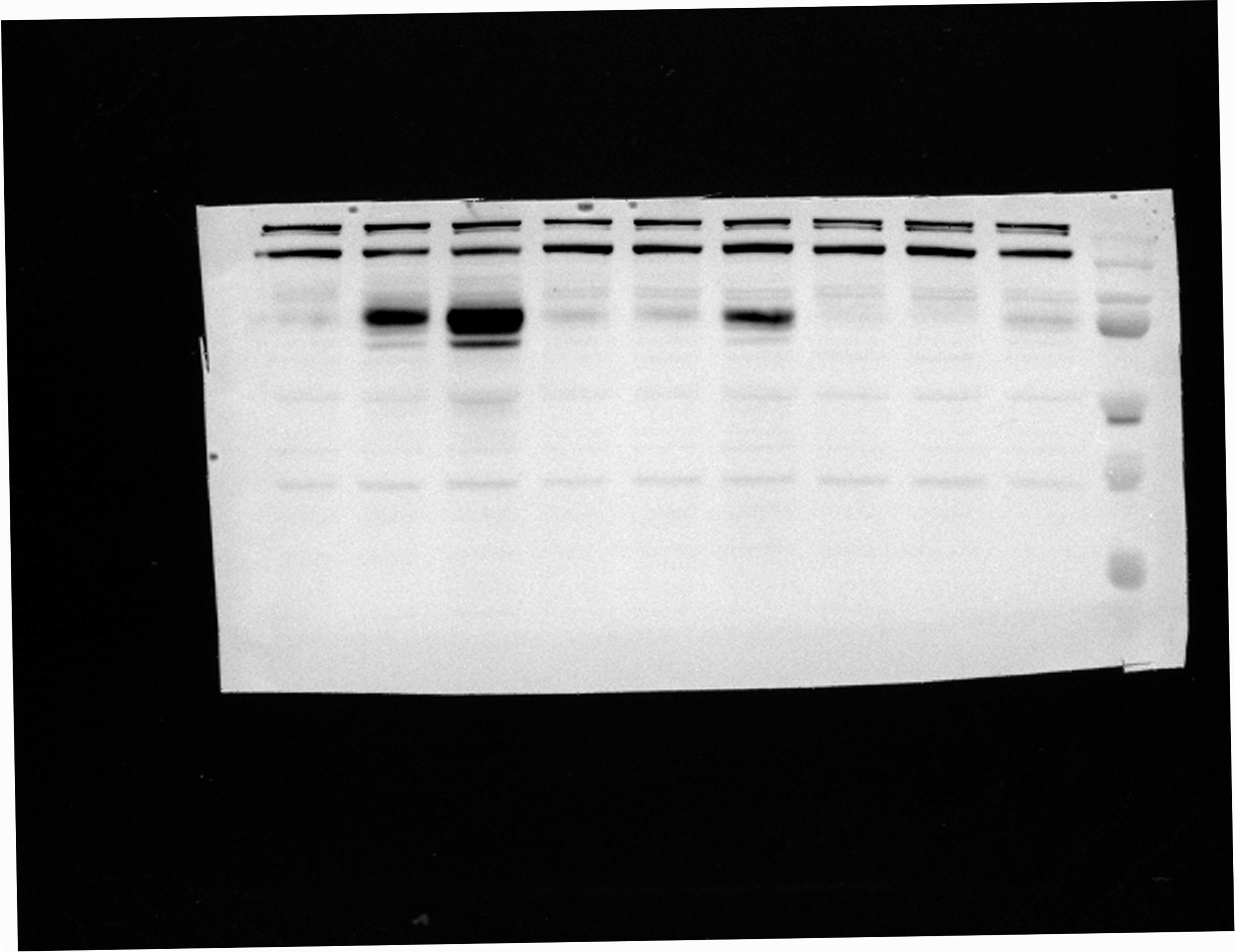

Supplement: Figure 5—source data 1. [file elife-71154-fig5-data1.zip › Figure 5-Source Data 1-ALPL/Figure 5-Source Data 1-ALPL blot raw.png]

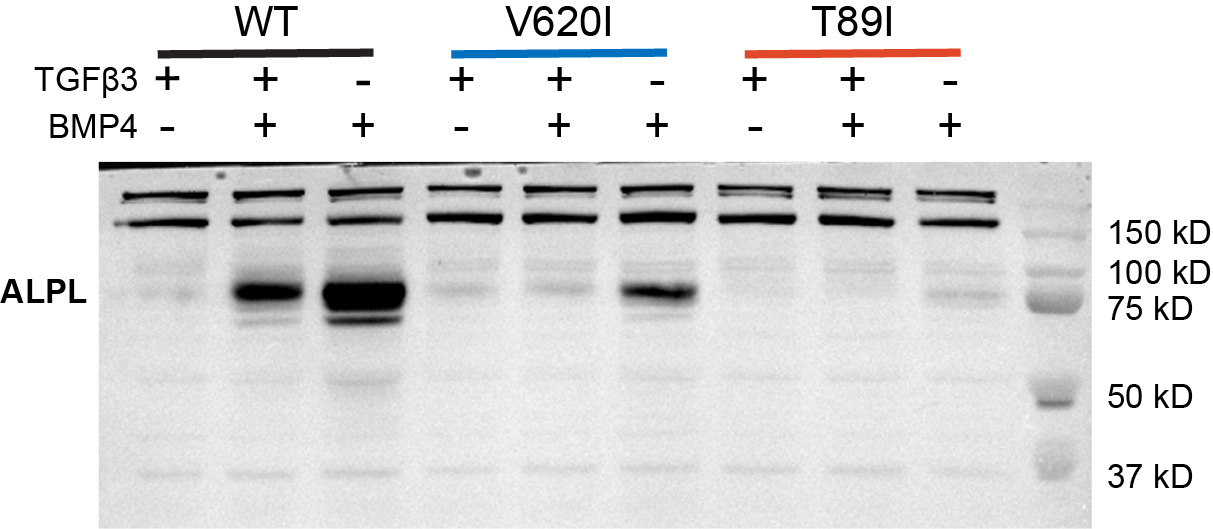

Supplement: Figure 5—source data 1. [file elife-71154-fig5-data1.zip › Figure 5-Source Data 1-ALPL/Figure 5-Source Data 1-ALPL blot with labels.tif]

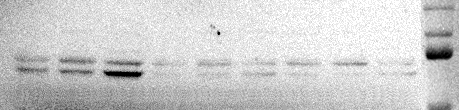

Supplement: Figure 5—source data 2. [file elife-71154-fig5-data2.zip › Figure 5-Source Data 2-COL10A1/Figure 5-Source Data 2-COL10A1 blot raw.tiff]

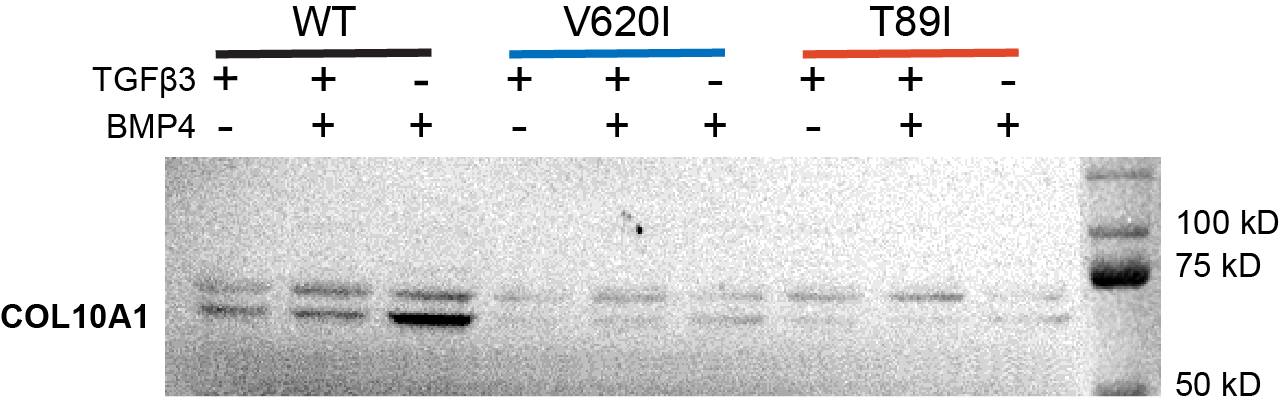

Supplement: Figure 5—source data 2. [file elife-71154-fig5-data2.zip › Figure 5-Source Data 2-COL10A1/Figure 5-Source Data 2-COL10A1 blot with labels.tif]

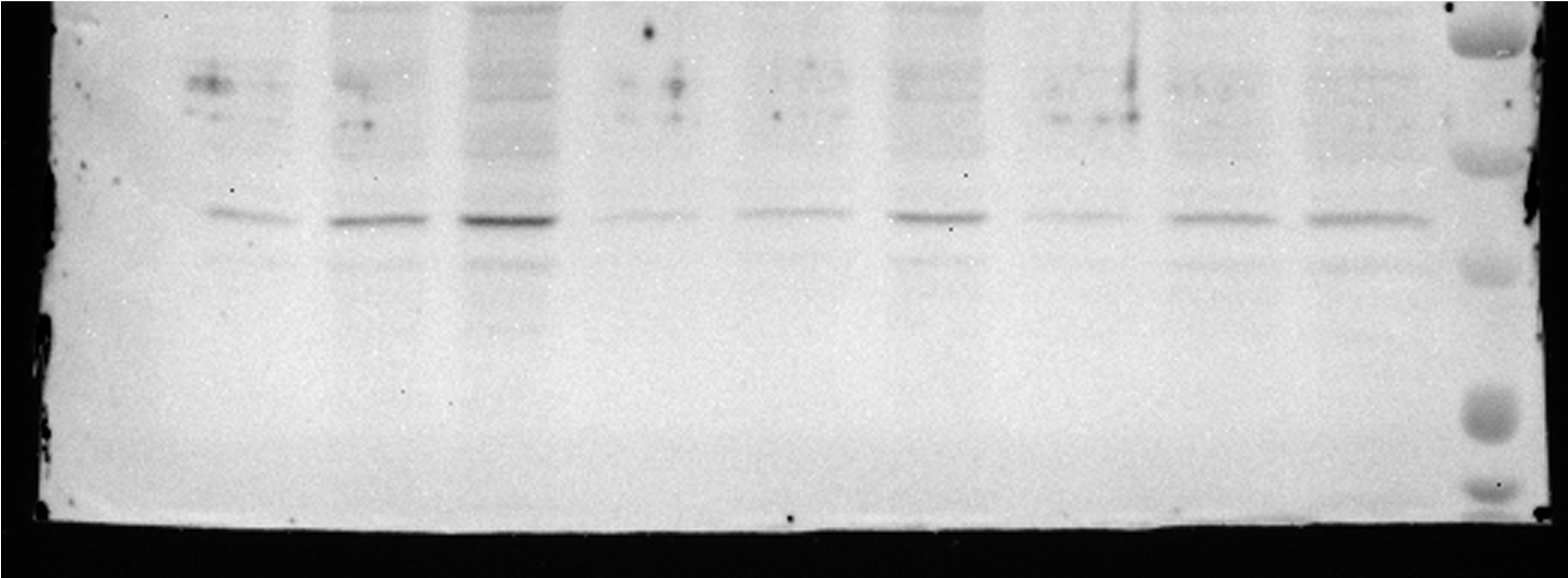

Supplement: Figure 5—source data 3. [file elife-71154-fig5-data3.zip › Figure 5-Source Data 3-IHH/Figure 5-Source Data 3-IHH blot raw.png]

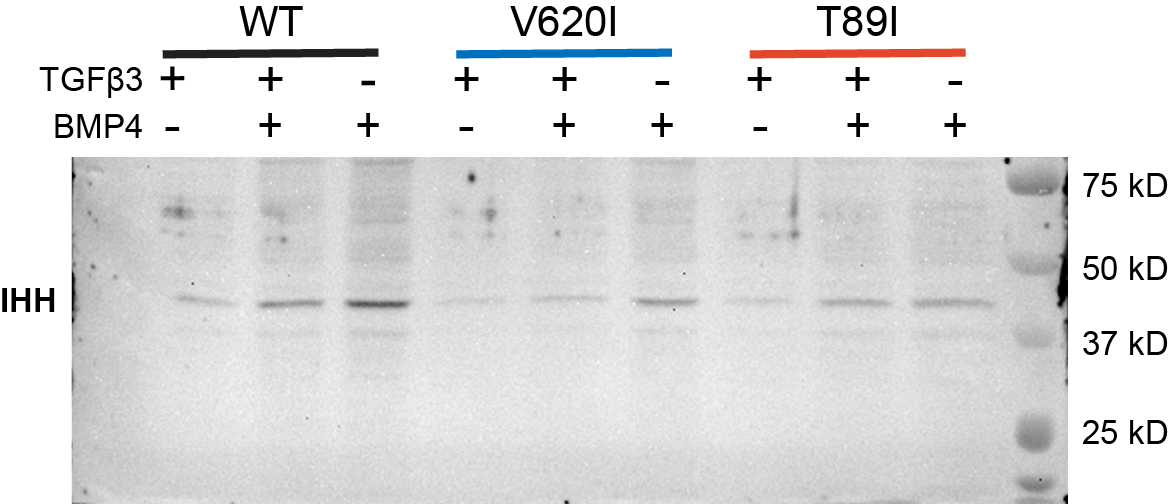

Supplement: Figure 5—source data 3. [file elife-71154-fig5-data3.zip › Figure 5-Source Data 3-IHH/Figure 5-Source Data 3-IHH blot with labels.tif]

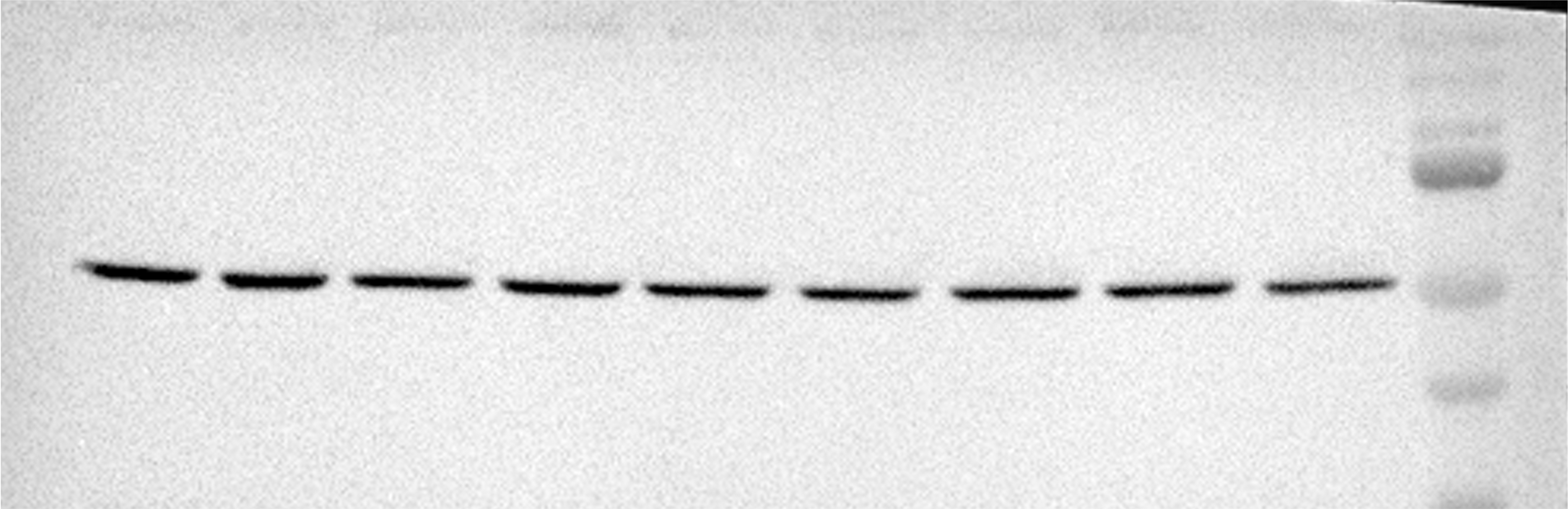

Supplement: Figure 5—source data 4. [file elife-71154-fig5-data4.zip › Figure 5-Source Data 4-MMP13/Figure 5-Source Data 4-MMP13 blot raw.png]

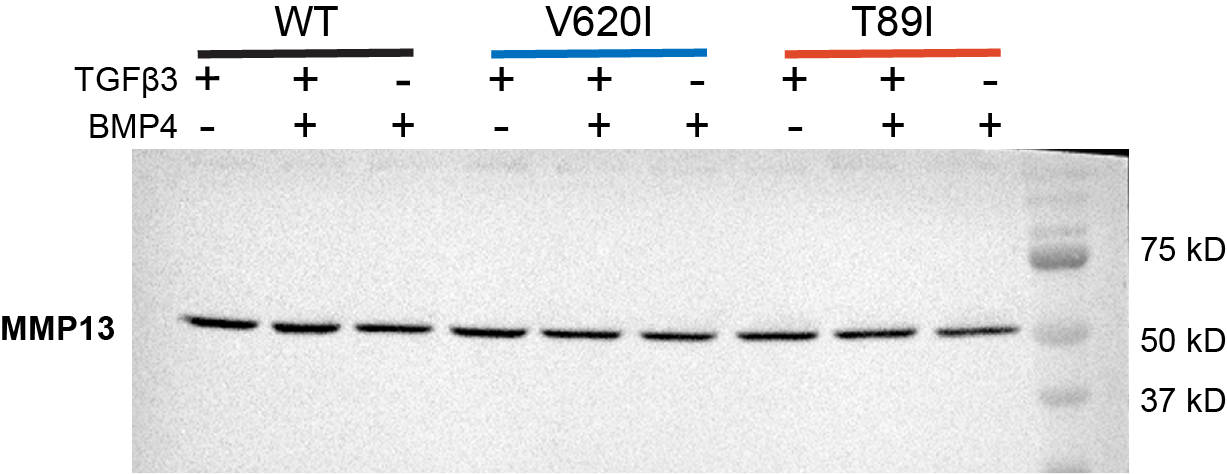

Supplement: Figure 5—source data 4. [file elife-71154-fig5-data4.zip › Figure 5-Source Data 4-MMP13/Figure 5-Source Data 4-MMP13 blot with labels.tif]

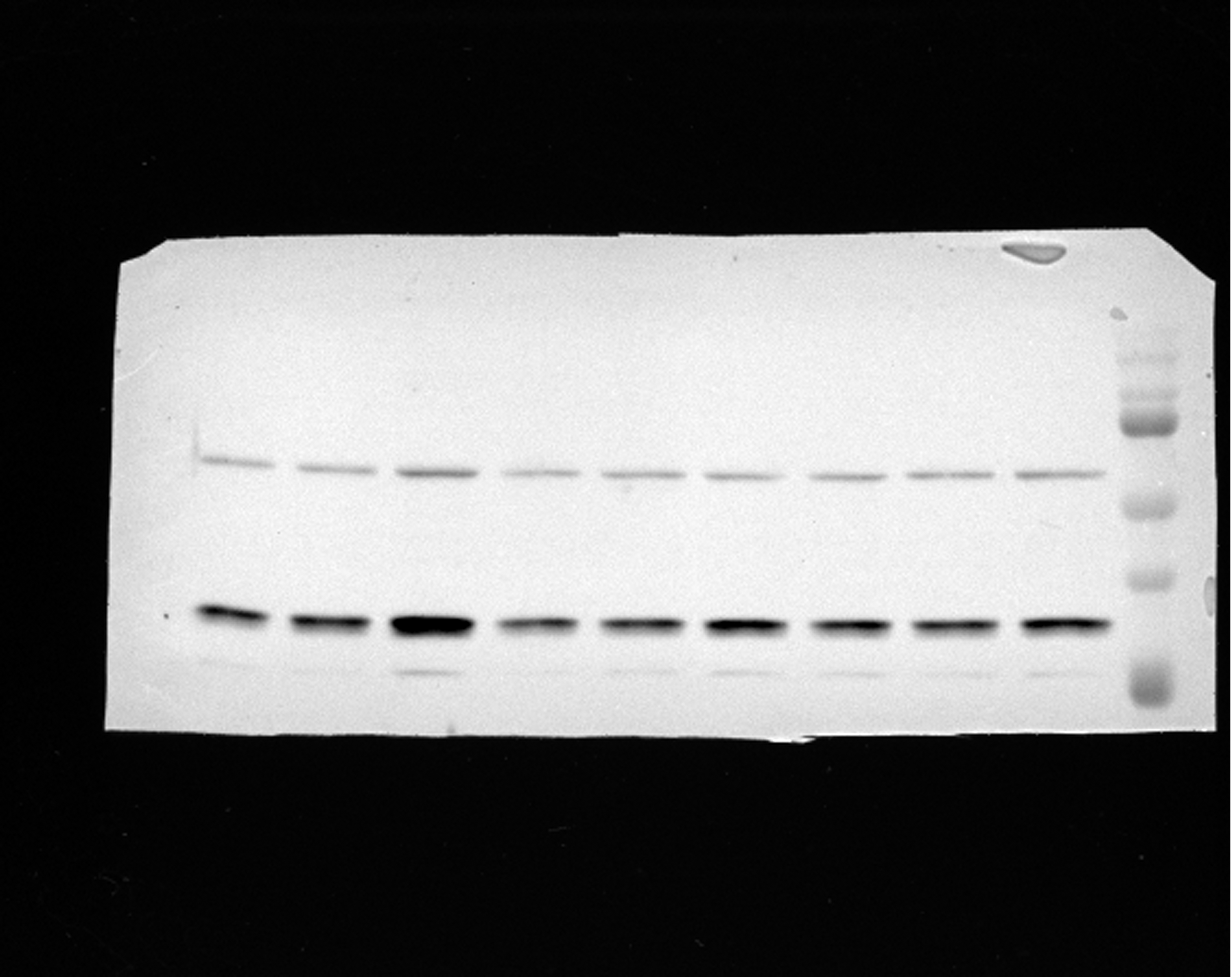

Supplement: Figure 5—source data 5. [file elife-71154-fig5-data5.zip › Figure 5-Source Data 5-RUNX2/Figure 5-Source Data 5-RUNX2 blot raw.png]

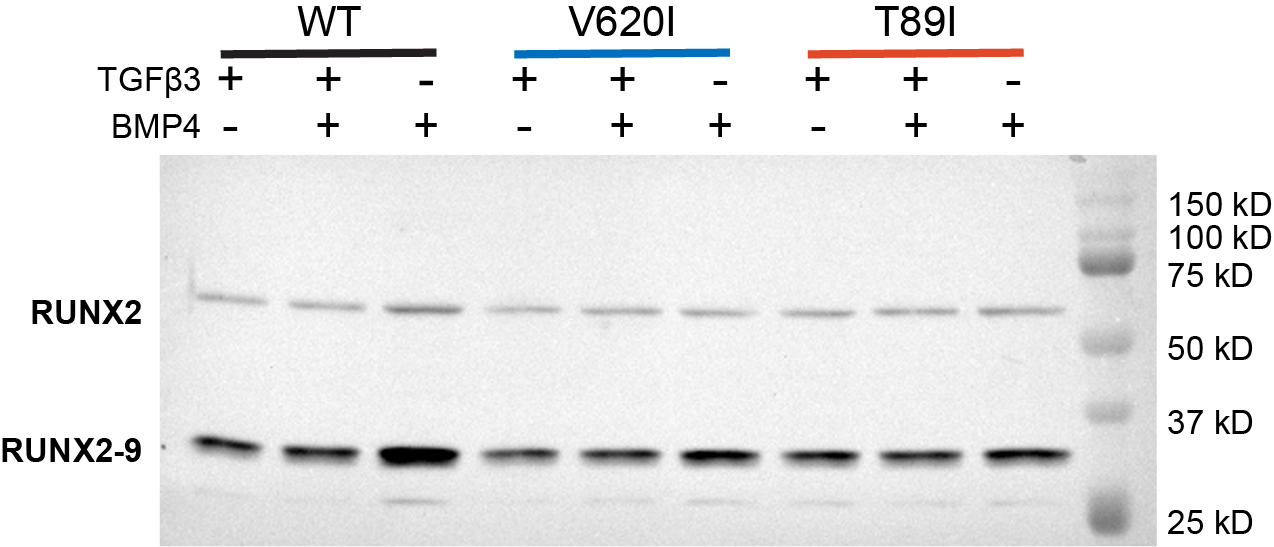

Supplement: Figure 5—source data 5. [file elife-71154-fig5-data5.zip › Figure 5-Source Data 5-RUNX2/Figure 5-Source Data 5-RUNX2 blot with labels.tif]

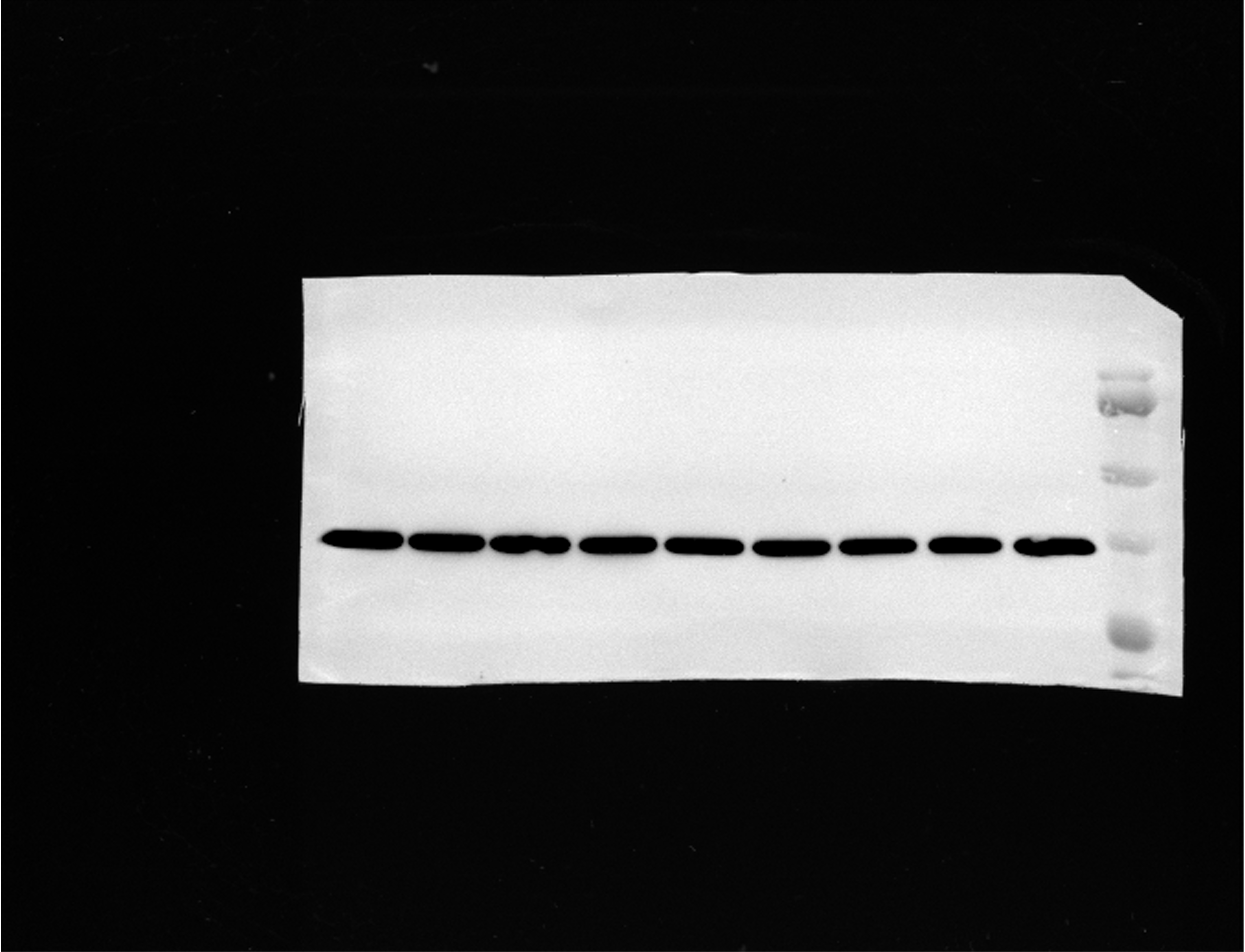

Supplement: Figure 5—source data 6. [file elife-71154-fig5-data6.zip › Figure 5-Source Data 6-GAPDH/Figure 5-Source Data 6-GAPDH blot raw.png]

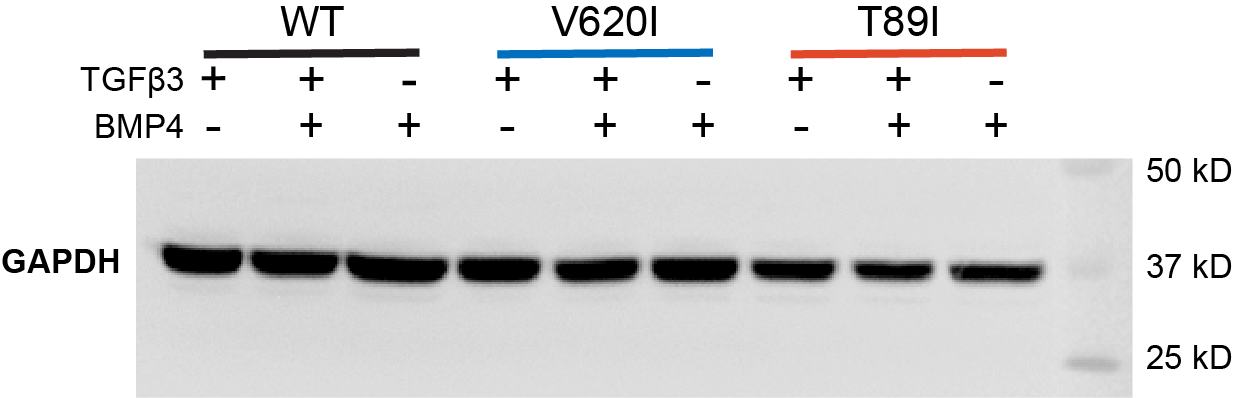

Supplement: Figure 5—source data 6. [file elife-71154-fig5-data6.zip › Figure 5-Source Data 6-GAPDH/Figure 5-Source Data 6-GAPDH blot with labels.tif]
